# Management of asthma patients during the COVID-19 pandemic: pathophysiological considerations to address the challenges

**DOI:** 10.1186/s43088-022-00204-4

**Published:** 2022-02-05

**Authors:** Tahani Tabassum, Ahsab Rahman, Yusha Araf, Md. Asad Ullah, Mohammad Jakir Hosen

**Affiliations:** 1 Biotechnology Program, Department of Mathematics and Natural Sciences, School of Data and Sciences, Brac University, Dhaka, Bangladesh; 2 Department of Genetic Engineering and Biotechnology, School of Life Sciences, Shahjalal University of Science and Technology, Sylhet, Bangladesh; 3 Department of Biotechnology and Genetic Engineering, Faculty of Biological Sciences, Jahangirnagar University, Dhaka, Bangladesh

**Keywords:** COVID-19, Asthma, Respiratory distress, Pathophysiology, Medications, Self-care, Telehealth, Risk, Management

## Abstract

**Background:**

The coronavirus disease 2019 (COVID-19) has become a serious global health issue, especially for people with pre-existing health conditions. Patients dealing with asthma are presumed to be at higher risk as COVID-19 may cause severe respiratory distress.

**Main body:**

From the initial stage of the pandemic, several clinical trials and studies have assessed the association between COVID-19 and asthma; however, no significant association was reported. This may be due to the fact that most of the asthma cases remained undiagnosed and overlapping respiratory features make it difficult to differentiate between these two diseases. The pathomechanism of the conditions and the immune response generated in response to the conditions suggest that the presence of any of the conditions is very likely to influence the presence or severity of the other condition. So far, no specific treatments are known for COVID-19; however, the use of plasma therapy and broad-spectrum antiviral drugs during the initial phase of the pandemic and widespread vaccination during the latter phase has given positive outcomes in reducing COVID-19 cases as well as disease severity.

**Short conclusion:**

Taking asthma as an increased risk factor for COVID-19 morbidity, this article aims to provide comprehensive insights into the risk and proper management of asthma patients during this COVID-19 pandemic. The common medications of asthma patients suppress their respiratory immune response that might facilitate cytokine storm in COVID-19 patients. Similarly, there are risks of viral-induced asthma exacerbations. Besides, different social issues such as shortage of medicines, SDOH, and delayed clinical trials put asthma patients through inconvenience. The primary focus at this point should be to reduce probable asthma attacks and severity to prevent hospitalization of asthma patients. Moreover, for better management of asthma patients maintaining an asthma action plan and healthy lifestyle, ensuring a nutritious diet, and developing self-management interventions can play a crucial role.

## Background

The pandemic coronavirus disease 2019 (COVID-19) caused by severe acute respiratory syndrome coronavirus-2 (SARS-CoV-2) has become a serious public health issue all over the world [1]. Until today, nearly 320 million people in 213 countries around the globe have been affected and 5,539,421 have died due to this disease [2].

The zoonotic virus SARS-CoV-2 generally infects the susceptible human body through nasal discharge, sneezes, coughs, or droplets of saliva from an infected person. Primarily, the virus targets the human respiratory system but often can target other organs causing multiple organ failures. Patients infected with COVID-19 have been found to have higher levels of plasma pro-inflammatory cytokines and an increased number of leukocytes [3, 4]. Although all categories of people are at risk of contracting the viral infection, people with pre-existing lung diseases like asthma, chronic obstructive pulmonary disease (COPD), and other comorbidities such as diabetes, hypertension, cancer, rheumatic diseases, and cardiovascular disorders appear to be more vulnerable to the disease severity and mortality [5, 6]. Among the COVID-19 infected elderly population, comorbidities associated with ageing and immunosenescence have also raised major concerns as such conditions are not only considered to increase susceptibility to the disease but also reduce the effectiveness of vaccines [7, 8]. Upon its emergence, COVID-19 had raised several burning questions including whether or not asthma patients are at increased risk during the pandemic. In this review, we have addressed the pathological aspects of asthma patients as well as discussed their probable management strategies during this pandemic.

## Clinical features of COVID-19 and asthma

### Symptoms and prognosis of COVID-19

During the initial four days of COVID-19 infection, patients may experience increased body temperature above 100°F with difficulty in breathing. Besides, patients may also experience mild muscle pain, constant fatigue, runny nose, and sore throat [9, 10]. During the next five to eight days, the patients may face greater difficulty in breathing, especially older people with pre-existing health conditions are likely to experience extreme discomfort. Sore throat and runny nose are two of the more common symptoms at this stage. If the condition deteriorates, the patient needs to be hospitalized [9–11]. During the late nine to fourteen days, patients may find it challenging to receive a sufficient amount of oxygen, a condition referred to as silent hypoxia [12]. As a result, patients can develop acute respiratory distress syndrome (ARDS), which can induce significant damage to the lungs. Abdominal pain and loss of appetite are also prevalent throughout this phase. Patients may even need to be transferred to the intensive care unit (ICU) during this phase [9–11]. In addition to the symptoms listed above, a patient may also encounter diarrhea, dyspnea, and some other symptoms as mentioned in Table 1[13–16].

**Table 1:** Common symptoms associated with COVID-19 infection

Symptoms	Percentage	References
Cough	59–82	[13–16]
Fatigue	44–70	
Headache	13.6	
Fever	83.99	
Diarrhea	10	
Sputum production	28–33	
Shortness of breath	31–40	
Myalgia	11–35	
Anorexia	40–84	

### Symptoms, causes, and classification of asthma

Asthma is a complex, multifactorial, and chronic non-communicable disease caused by a combination of genetic, environmental, and occupational factors. It is characterized by inflammation and narrowing down of the oxygen-carrying airways [17–20]. Inflamed air passages are extremely sensitive to environmental triggers, producing extra mucus and increasing the difficulty in airflow to the lungs. In response of the airway to these triggers, a person may experience an "asthma flare-up" or "asthma attack" [20], characterized by intensive coughing, wheezing, chest tightness, and breathing difficulty [21]. Asthma displays heterogeneity in clinical features due to multiple drivers such as age, sex, race, socioeconomic status, and environment [22]. According to the World Health Organization (WHO), symptoms of asthma can be pointed as recurrent attacks of breathlessness, wheezing, sleeplessness, fatigue, and reduced activity [18, 23]. Although several epigenetic considerations determine asthma susceptibility and severity, this condition has been reported to be more prevalent in children and young adults, putting this age group at increased risk.

Based on severity, asthma can be divided into four categories or stages: mild intermittent, mild persistent, moderate persistent, and severe persistent (Table 2) [24–27]. In each of these stages, a gradual drop-in respiratory rate and lung function can be observed.

**Table 2:** Correlation between asthma types and respiratory system function

Types	Respiratory rate (based on age)	Lung function	Symptom frequency	Severity	Night time awakening	Pulse rate	References
Mild intermittent	No change or slightly increased	Almost normal	May happen 2 days a week	Does not affect regular activities	2 or fewer times each month	< 100	[25–27]
Mild persistent	Increased	80% of normal or greater	More than twice a week	Minor impact on regular activities	3 or 4 times	100–120	
Moderate persistent	Increased often > 30	Between 60–80%	On a daily basis	Limits daily activities somewhat	More often than once a week	> 120	
Severe persistent	Normal or decreased	Less than 60%	Symptoms will arise throughout the day	Significantly limits daily activities	Every night	Bradycardia	

### Overlapping features between COVID-19 and asthma

Proper understanding of distinct clinical features of COVID-19 and asthma is crucial to avoid any misinterpretation and ensure an accurate diagnosis. Patients of both COVID-19 and asthma show overlapping features including coughing, fatigue, and shortness of breath, which makes diagnosis and proper management of both diseases quite challenging. However, literature studies reveal that some symptoms such as diarrhea, myalgia, loss of smell and taste, headache, and prolonged fever are the distinctive features of COVID-19 and are not evident in asthma. On the other hand, an asthma patient may experience wheezing, which is not evident in COVID-19. The comparative features of COVID-19 and asthma are presented in Table 3 [28–31].

**Table 3:** Overlapping symptoms between COVID-19 and asthma

Symptoms	Presence in COVID-19	Presence in asthma	References
Fever	Yes (very often)	No (rarely)	[28–31]
Wheezing	No	Yes	
Weariness	Yes	No	
Zoonotic origin	Possibly yes	No	
Sore throat	Yes	No	
Muscle pain	Yes	No	
Diarrhea	Yes	No	
Myalgia	Yes	No	
Loss of smell or taste	Yes	No	
Confusion headache	Yes	No	

## Pathophysiology

### Pathophysiology of COVID-19

**Fig. 1:**
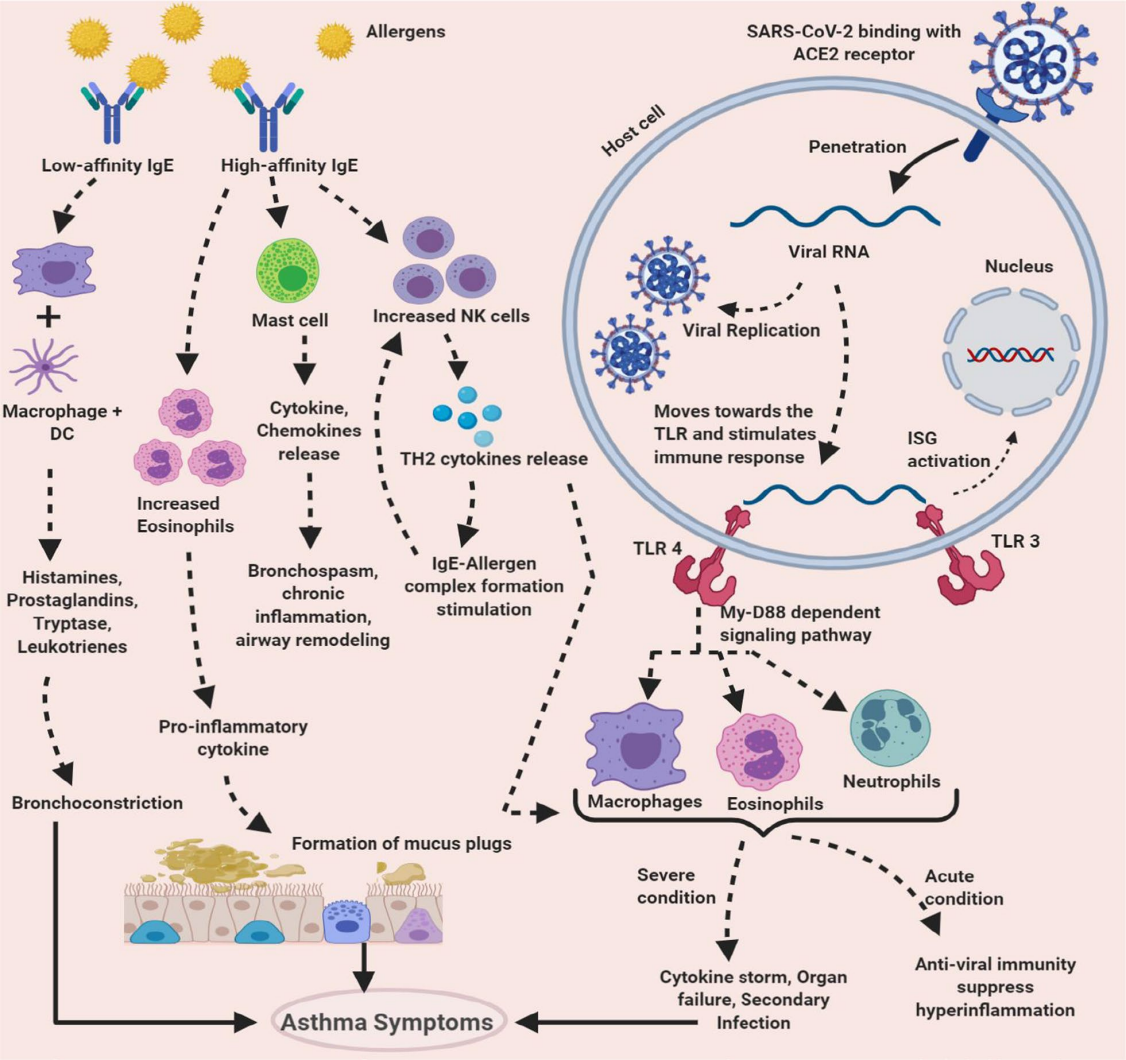
The pathophysiology of SARS-CoV-2 and allergic asthma. SARS-CoV-2 binds to the ACE2 receptor and injects its RNA inside the host cell. The dsRNA triggers TLR3 and TLR4 leading to ISG activation and recruitment of immune cells. In severe conditions, the immune cells lead to cytokine storms which can trigger asthma symptoms. On the other hand, allergens can form complexes with high-affinity IgE and low-affinity IgE, which can trigger immune cells to release cytokines and other mediators that lead to the formation of mucus plugs, bronchoconstriction, airway remodeling, and other asthma symptoms. In SARS-CoV-2 patients with asthma, the recruitment of immune cells and the release of cytokines from both pathways can lead to severe organ damage and other harmful effects. This picture was created with BioRender and downloaded with a premium subscription

SARS-CoV-2 follows a lytic replication cycle and employs the host metabolic machinery to replicate itself. The virus invades human respiratory host cells in five steps: attachment, penetration, biosynthesis, maturation, and release (Fig. 1) [32, 33].

SARS-CoV-2 enters the host cell using angiotensin-converting enzyme-2 (ACE-2) receptors, which are highly expressed in the apical mucosal membranes of the lung, kidney, heart, ileum, small intestine, and bladder of mammals [34]. The virus primarily attacks the ACE-2 receptors in the respiratory tract using their Spike protein, but it has the potential to invade other host cells that can drive multi-organ damage. ACE-2 is also used by SARS-CoV to invade host cells, but the affinity appears to be much more robust in SARS-CoV-2 due to the presence of a unique furin cleavage site in their S-protein. Besides, reports imply that the virus can associate with a transmembrane glycoprotein named CD147 that is highly expressed among tumor tissues and pathogen-infected cells, revealing another novel route for the virus to invade the host cells [35]. Once the virus releases its genomic RNA into the host cytoplasm, the dsRNA evokes an immune response by triggering toll-like receptors (TLR), such as TLR-3 and TLR-4. TLR-3 stimulates type-1 interferon (IFN) by signaling pathway cascade, which in turn drives the expression of interferon-stimulated genes (IFGs). On the other hand, TLR-4 activates pro-inflammatory cytokines and recruits immune cells to the site of infection (Fig. 1) [36, 37]. After digestion, the antigenic parts of the virus are presented to the T cells in the secondary immune organs through the antigen-presenting cells (APCs), and the T cells start to divide [36]. This process is crucial for viral clearance but may sometimes lead to an over-activated inflammation known as "cytokine storm".

Evidence from severe COVID-19 patients suggests the mechanism of "cytokine storms" are due to exceptionally high levels of interleukin (IL)-1β, IL-1Rα, IL-7, IL-8, IL-9, IL-10, basic fibroblast growth factor (FGF), granulocyte-colony stimulating factor (G-CSF), granulocyte–macrophage colony-stimulating factor (GM-CSF), IFN-γ, interferon-γ-inducible protein (IP-10), monocyte chemo-attractant protein (MCP-1), macrophage inflammatory protein 1 alpha (MIP-1α), macrophage inflammatory protein 1 beta (MIP-1β), platelet-derived growth factor (PDGF), tumor necrosis factor (TNF-α), and vascular endothelial growth factor (VEGF) [38]. Critical patients have also exhibited deficient levels of natural killer (NK) cells, memory T cells, T regulatory cells, along thrombocytopenia and lymphocytopenia [39]. Besides, the lymph node and spleen atrophy indicate an impaired immune response [38]. The two probable causes for such extensive damage to the immune system might be through direct viral attack or by a cytokine storm. Direct viral infection on the immune cells is a possible phenomenon, as a limited number of ACE-2 receptors are found in dendritic cells (DCs) and alveolar macrophages.

### Pathophysiology of asthma

The application of new molecular and biological techniques allowed the evaluation of phenotypic aspects and complex interacting pathways in asthma, revealing a variety of factors incurring the recurrent airflow limitation of the condition [40].

Bronchoconstriction is a prominent feature in asthma, which leads to the narrowing of the airway. Allergy-specific immunoglobulin E (IgE) reacts to stimuli like allergens and irritants, mediating the release of histamine, tryptase, prostaglandins, and leukotrienes that causes bronchoconstriction (Fig. 1). Some nonsteroidal anti-inflammatory drugs may also evoke mediator release, resulting in bronchoconstriction [41]. The mechanism of such IgE-dependent and non-IgE-dependent pathways is not well described but appears to be associated with underlying inflammation.

Asthma is mostly mediated by type-2 immune response comprising type-2 B cells, group 2 innate lymphoid cells, helper T cells, NK cells, basophils, eosinophils, mast cells, and NKT cells [42]. Evidence suggests tremendous expression of TH2 cytokine in asthmatic patients that explain the overproduction of high-affinity IgE molecules, eosinophils, hyperresponsiveness in the air tract, increase in NK cells, and subsequent decline in regulatory T cells [43].

Besides, mucosal mast cells in the airway are activated by the allergens. These immune cells release cytokines and other mediators to promote inflammation and acute bronchospasm. The severity of this situation correlates with an increased number of eosinophils, releasing a wide range of pro-inflammatory cytokine and inflammatory enzymes [44]. However, the role of eosinophils in asthma is being reconsidered. A recent study shows an anti-IL-5 treatment that can reduce the number of eosinophils in the air tract cannot manage the disease [45]. Neutrophils also increase in the airway of severe asthma patients, but their pathophysiological role remains uncertain [46]. All these inflammatory cells and mediators like chemokines and cytokines do not only affect the airway smooth muscles but also in severe cases, lead to airway dysfunction, obstructive lesions, and other injuries [47]. As the inflammation worsens, factors like increased mucus secretion, edema, and the formation of inspissated plugs cause hyperplasia of the airway smooth muscles and airway remodeling, rendering the person less responsive to drug or therapy. These physiological changes lead to the recurrent occurrence of wheezing, dry cough, and shortness of breath [48].

## Prevalence of asthma in COVID-19 patients

Numerous studies have claimed that COVID-19 is not a significant threat to asthma patients, while some research has shown that asthma patients might be under increased risk of contracting COVID-19 [49]. According to the Centers for Disease Control and Prevention (CDC), COVID-19 can be perilous to people with moderate to severe asthma [15]. UK Biobank-based study estimated that non-allergic asthma can increase the risk of SARS-CoV-2 infection by 48% [50]. Another observational study reports that 14% of the 16,749 hospitalized patients with COVID-19 had asthma [51]. A large cohort English study had found that asthma could increase the risk of in-hospital death from COVID-19 significantly [52, 53]. CDC study in March 2020 on hospitalized patients of the USA has reported that 27.3% of the hospitalized COVID-19 patients with the age range of 18–49 years had a history of asthma [28, 54]. 13.2% of the hospitalized COVID-19 patients aged 50–64 years and 12.9% of the hospitalized COVID-19 patients aged 65 and more have asthma [54, 55]. It is thereby certain that people in 18–49 years might be susceptible to have asthma during the viral pandemic. Although increasing evidence shows that children are less likely to be affected by COVID-19 [56], there is a possibility of viral-induced asthma exacerbation if a child with asthma is infected with COVID-19 [54, 57]. Evidence in the USA showed 3 out of 24 critically ill COVID-19 patients in Washington State had pre-existing asthma [58].

On the contrary, several studies have debunked the idea of asthma patients being at increased risk during the COVID-19 pandemic. The prevalence of asthma patients with COVID-19 is most likely to be lower than expected from population levels based on the data from both USA and China [59]. Data from Korea also disapprove of the relevancy of asthma as comorbidity [60]. A study from 552 hospitals across 30 provinces in China revealed that asthma was not a pre-existing condition [11, 61]. In another cohort, only 0.9% of COVID-19 patients were reported with asthma [62]. Moreover, The New York Times has reported that only 5% of the COVID-19 patients who have died had asthma in New York [63].

Moreover, asthma is not listed in the top ten comorbid health conditions present in COVID-19 patients [64]. Many of the studies have not reported chronic pulmonary diseases like asthma, and bronchiectasis in their COVID-19 cohort [65, 66]. Since it is difficult to differentiate between COVID-19 infection and asthma, this underrepresentation and underreporting can be the result of the poor diagnosis. So, there is a possibility that asthma patients could still be at increased risk during the pandemic. To understand the relationship between asthma and COVID-19 infection comprehensive diagnosis and efficient differentiation between both conditions is mandatory.

## Impacts of asthma in COVID-19 infestation

### Correlation of asthma and COVID-19 pathomechanism

Although there is mixed opinion on the impact of asthma in COVID-19, some of the reported results together with the previous SARS-CoV outbreak suggested that asthma may have a potential impact on the susceptibility and severity of COVID-19 due to the impaired immune response against viral infection in asthmatic patients [67]. Since the pathological aspects of all the human coronaviruses are similar, it is assumed that predisposal to allergens might weaken the anti-viral response in asthmatic patients and increase their susceptibility to COVID-19 [42].

As of now, studies suggesting a probable association between asthma and ACE2 expression are insufficient; however, there are several studies on the impact of lessened ACE-2 expression in asthmatic COVID-19 patients. These studies suggested that the expression of ACE2 is most likely regulated reciprocally by IFN levels and type-2 cytokines in the blood, especially in allergic asthma [68]. This could partly explain the reason why asthma was not associated with extensive damage and poor consequences such as respiratory failure and death. Besides, ACE-2 receptors might be down-regulated due to controlled allergen exposures in asthmatic patients [69]. Studies show that type-1 IFN and type-2 IFN regulate ACE-2 expression in cells but due to their deficiency in asthma expression of these receptors will be low, partially limiting the viral invasion on target cells. It is also possible that an elevated amount of type-2 cytokines in asthma may counteract the pathogenesis of the virus and accumulation of pro-inflammatory cytokines [47]. Another vital inflammatory T2 cytokine that is accountable for the ACE‐2 down‐regulation in SARS-Cov-2 patients is IL‐13 [70]. These shreds of evidence suggest a probable protective role of type-2 immune responses in asthma patients against COVID-19 infection.

Eosinophil plays a central role in asthma and has a potential role in antiviral host defense [42]. A recent study demonstrated that 78% of the hospitalized asthmatic patients had no detectable levels of eosinophil during admission, presenting severe eosinopenia as a prognostic marker among the critical asthmatic COVID-19 patients [71]. It is suggested that eosinopenia is the result of rapid immune cell destruction caused by direct SARS-Cov-2 targeting or cytokine storm. However, eosinophilia in asthma may counter this condition in COVID-19 patients, which might provide a substantial benefit to asthmatic patients [47].

Human coronaviruses can inhibit IFN signaling, a significant pathway to activate the host innate immune system [47]. Several studies on severe atopic patients have demonstrated a defective production of IFNs by plasmacytoid DC and epithelial cells [72] that may be correlated to delayed and insufficient anti-viral activity and may lead to poor outcomes in asthmatic patients.

### Asthma medication and COVID-19 infestation

A significant dilemma for COPD patients is whether or not to continue consuming their regular prescribed asthma medications during the pandemic. Since SARS-CoV-2 is a respiratory virus, patients are concerned about their prescribed medicines [67].

#### Nebulizer and inhaler

Nebulization therapy directly transfers drugs in the form of mists or droplets to the site of action in the lung, but a portion of this aerosol might return to the reservoir tube [73], increasing the risk of transmission of COVID-19 [74]. These nebulized droplets might contaminate the device if it is used by a COVID-19 patient, further increasing the risk of spreading the virus [28]. Studies show that about 50% of these generated aerosols can persist in the air for hours that increasing the risk of transmission to susceptible bystander hosts [73].

The National Institute for Health and Care Excellence (NICE) states that the fluid inside the nebulizer chamber will not have any virus particles from patients. Still, it is recommended to avoid the use of nebulizers and consider alternatives such as metered-dose inhalers (MDIs), dry powder inhalers (DPIs), or spacers. However, asthma patients with COVID-19 might be allowed to use a nebulizer under three conditions: (1) if they are suffering from life-threatening conditions; (2) if they have an inadequate response to the alternatives while using minimal oxygen flow rate to drive the nebulizer; and (3) if the patient is unable to manage alternatives other than nebulization [52].

#### Inhaled corticosteroids

Inhaled corticosteroids (ICS) are the most effective and comprehensive asthma controllers. Even in low doses, the drug can suppress inflammation and reduce inflammation-mediated lung injury [75].

There is much confusion regarding the use of ICS during this pandemic. Some studies have revealed that the use of ICS alone or in a combination of bronchodilators can have some positive impact on reducing viral infection [75]. However, some other studies showed an increased risk of pneumonia, the possibility of change in the lung microbiome, and even delayed viral clearance in the lower respiratory tract due to the use of ICS [76].

There is some evidence of ICS being helpful, such as pretreatment of human respiratory epithelial cells with a combination of medications like glycopyrronium, formoterol, and budesonide may inhibit SARS-CoV-2 replication and reduce cytokine production [77, 78]. A study also suggested that ICS therapy can reduce ACE-2 and transmembrane protease serine 2 enzyme (TMPRSS2) gene expression from sputum [79], which may protect against COVID-19 alongside providing therapeutic benefits for asthma. Besides, ICS is known to reduce pro-inflammatory cytokines and increase anti-inflammatory cytokines, which can exert some protection during the earlier stages of COVID-19 infection. In vivo studies of inhaled ciclesonide in SARS-CoV-2 replication show inhibition of its cytopathic activity [78], suggesting that corticosteroids may help treat COVID-19 but the mechanism is still unclear [80].

#### Oral corticosteroids

Based on previous experiences with Influenza, SARS, and the MERS epidemic, organizations like WHO and CDC have recommended stopping the use of oral corticosteroids (OCS) to treat COVID-19 patients. It is highly suspected that the use of OCS can prolong viral replication, complicate viral clearance, increase the risks of ventilation, cause secondary infections, and raise mortality rates [15].

Corticosteroid prednisone, a widespread recommended asthma medication, is not suspected to compromise the immune system or increase susceptibility to COVID-19 infection when used in a brief course. Thus, asthma patients without experiencing COVID-19 symptoms should continue to treat their exacerbations using the drug. However, if the patient is suspected of being infected by the virus, prednisone cannot be considered safe as it might prolong viral replication [81]. Front line physicians from the Chinese Thoracic Society have set up some principles to consider before suggesting oral corticosteroids during this pandemic which includes: the beneficial and harmful effects should be evaluated, the drugs should be used carefully in COVID-19 patients with pneumonia, cautious use of OCS in patients with hypoxemia or who have been using OCS for a chronic disorder like asthma, low to-moderate dose (≤ 0·5–1 mg/kg per day, in short duration ≤ 7 days) [82].

On the other hand, a report by WHO shows dexamethasone, an OCS used for asthma and some other complications, can be lifesaving for severe COVID-19 patients. The WHO Director-General stated that it was the first treatment to be shown to reduce mortality in patients with COVID-19 using ventilation support [83].

#### Biopharmaceutical agents

Biologics like anti-IgE and anti-IL5 monoclonal antibodies are very useful in reducing the frequency of asthma exacerbations. Studies including long-term follow-ups indicate no signs of the impaired immune system or increased risks of viral infection due to the use of these biologics [81]. The use of anti-IgE and anti-IL5 may even protect asthma patients from the risks of viral-induced exacerbations [84].

Recent studies have reported that blocking IgE can reduce the possibility of being infected by respiratory viruses because IgE is responsible for weakening anti-viral response in asthma patients. Besides, anti-IgE monoclonal antibodies can upregulate IFN-α signaling in dendritic cells and decrease viral infection duration along with levels of IL-33 that induce pro-inflammatory cytokines [84]. All these compliances suggest a probable positive effect of omalizumab on SARS-CoV-2 susceptibility and severity.

#### Allergen immunotherapy

Allergen immunotherapy (AIT) has been deemed as one of the best approaches to "personalized medicine" in allergic diseases [85]. AIT can modify the immune response by altering the IgE spectrum. It has a long-term effect, which is not found in other allergy treatments [86]. AIT modifies T and B cell response, causes degranulation of allergy effector cells, and maintains regulatory B and T cells. Regulatory T cells can release inhibitory cytokines to suppress TH2 responses, playing an essential role in controlling cytokine storm, inflammation, and limiting lung tissue damage [87]. Considering the role of AIT to suppress cytokine storms, AIT may exhibit some protection against the COVID-19.

### Impact of drugs and vaccines used for COVID-19 treatment in asthma patients

During the initial stage of the pandemic, programs established by FDA such as expanded access (EA) and emergency use authorization (EUA) allowed clinicians to gain access to investigational therapies to find any drug effective in reducing COVID-19 severity [88].

Remdesivir, an inhibitor of the viral RNA-dependent RNA polymerase, was the most widely used drug to treat COVID-19 patients due to its promising outcome against the SARS-COV-1 and MERS-CoV [89–91]. Clinical trials aimed at evaluating the efficacy of the drug showed shorter recovery time of hospitalized COVID-19 patients compared to the placebo group [91]. Since this drug targets the RNA polymerase that is unique to certain RNA viruses, it is expected that this drug does not have any impact on asthma, although there are controversies surrounding clinical trials assertive of such a claim [92].

Chloroquine (CQ) and hydroxychloroquine (HCQ), two of the synthetic drugs used in the treatment of acute attacks of malaria, can inhibit glycosylation of the ACE-2 receptor and interrupt the binding of SARS-CoV-2 to the host cell [93]. Besides, both of these drugs can also increase endosomal pH to inhibit viral fusion with the host membrane and block the transport of SARS-CoV-2 from early endosomes to endolysosomes preventing viral genome release [94, 95]. Since both of the drugs are anti-inflammatory agents, the administration of these drugs is expected to bring positive outcomes in asthma patients. In some cases, CQ is also used as a steroid-sparing agent in severe asthma patients [96], suggesting no major harm of these medications in asthma. Although there are studies confirming that CQ and HCQ have antiviral activity on SARS-CoV-2 in vitro [97], the effectiveness of these synthetic molecules and these being devoid of toxicity are still questioned by a portion of the scientific community [98].

The anti-inflammatory drug azithromycin has been widely evaluated for its efficacy against COVID-19 [99]. The immunomodulatory property of the drug can significantly downregulate cytokine production, maintain epithelial cell integrity, and prevent lung damage in COVID-19. The use of this drug has been associated with a reduction in mortality and severity of SARS-CoV-2 infection [100]. Considering its immunomodulatory potentials, it is also considered to be a safe medication for treating asthma exacerbations and poses no potent threat to asthma patients being treated for COVID-19 with this drug.

There is an ongoing debate regarding the possibility of worsening asthma symptoms while receiving the COVID-19 vaccines; however, no evidence to support such a hypothesis exists as of now. The effect of COVID-19 vaccination in asthma patients is entirely unexplored, raising concerns regarding the probable long-term effect such vaccines can induce in asthmatic patients. There are also concerns that asthma might increase the chances of allergic reaction to the COVID-19 vaccines; however, no such evidence has been found.

The rates of allergic reactions to COVID-19 vaccines are extremely low and there is no evidence of asthma patients being more susceptible to such allergic reactions. As per a weekly report of CDC, among the recipients of the Pfizer vaccine, 11.1 out of 1 million doses reported the case of anaphylaxis, the most severe type of allergic reaction. The rate of Moderna-induced anaphylaxis was 2.5 per 1 million doses. Among the 8 million doses of the Janssen vaccine, 4 anaphylaxis cases could be reported [101, 102].

Some studies have reported that mRNA vaccines are likely to induce IFN-1 production [103]. Hyperactive IFN-1 production has also been reported in viral-induced asthma exacerbations [104], indicating a probable negative impact of mRNA vaccines on asthma patients. However, further confirmation is required for such claims.

As of now, it is suggested that asthma patients should receive the full doses of COVID-19 vaccines if no significant side effect is reported after the first dose of the vaccine. According to the American College of Allergy, Asthma, and Immunology (ACAAI), even asthma patients prescribed with ICS or OCS must receive the vaccine if no severe side effect is reported [105].

## Challenges of asthma patients during the COVID-19 outbreak

### Shortage of medications

The current pandemic has severely affected the production and supply chain of medicine. Besides, stocking medicine much more than needed is creating artificial scarcity and increasing price [106]. In some cases, asthma medications like albuterol are being employed in COVID-19 treatment, furthering a decrease in the supply of this essential drug [107]. Previously salbutamol nebulizer solution was commonly used in hospitals, but due to the risk of spreading of virus through nebulization, the practice shifted to albuterol inhalers [52]. Due to the shortage of this commonly used asthma controller, patients are more likely to get severe asthma attacks and may even need to be hospitalized, which increases the risk of viral contamination.

### Impact of social determinants of health

Financial distress, physical environment, food and housing insecurity, and inability to access health care services, all these social determinants of health (SDOH) are being influenced due to the current pandemic situation. Especially for low-income families with no permanent job security or insurance benefits, it is difficult to maintain necessary physical distancing as there are concerns regarding food arrangements for the family. In such situations, taking care of an asthma patient becomes challenging, as they cannot afford to stockpile medicines or access telehealth programs [108]. Although some of these SDOH cannot be improved, proper planning and care may help reduce some of the impacts, such as reducing second-hand exposure to smoke, avoiding asthma triggers, and maintaining good hygiene [28, 109].

### Interference to care

Medication adherence in asthma includes a variety of factors including visits to the health care provider and routine follow-ups. The interaction between health care providers and asthma patients may boost their treatment adherence and eventually have a positive effect on asthma control [110]. Due to the pandemic, most of the follow-up visits of asthma patients have been either canceled or transferred to virtual visits, which may worsen asthma control and lead to frequent asthma attacks or exacerbation events.

### Virus-induced asthma exacerbations

Asthma exacerbation is defined as a reduction in the forced expiratory volume of more than 20% from baseline, or a decrease in peak expiratory flow of > 30% from baseline for two consecutive days at any time frame during the course of treatment [111]. It is also described as an emergency respiratory attack that is severe and needs immediate attention or else might lead to life-threatening situations. Viral infections are reported to cause ~ 60–70% of asthma exacerbations [112]. Although there are few reports of viral-induced asthma exacerbation during the SARS and MERS epidemic, it is yet undefined in the COVID-19 pandemic [113, 114].

### Asthma diagnosis and monitoring

Diagnosis of asthma follows a critical analysis of patient history as well as physical, biochemical, and radiological examinations, which can be difficult in this current pandemic [115, 116]. To monitor asthma during this pandemic, several questionnaires based on online platforms have been established. These platforms can help monitor asthma without the need to visit hospitals and predict reasonable asthma control [52]. However, there can still be barriers to skills and resources, limiting access to such online services. It is also recommended to keep a peak flow meter in the house and maintain a peak flow diary to keep a better record of asthma.

## Strategies to ensure better management of asthma during COVID-19 pandemic

### Telehealth

Telecommunications and virtual patient encounters can be viable alternatives to in-person checkups during this SARS-CoV-2 pandemic. Asthma and several other COPD patients can be diagnosed using automated computer-based methods. Researchers have suggested the diagnosis of asthma and COPD using artificial neural networks (ANNs), fuzzy logic (FL), and an expert system (ES). Based on these suggestions, some facilities in combination such as a portable spirometer that can be connected to a mobile phone via Bluetooth, a well-developed android operating system installed in that mobile phone, and an expert system stored on the mobile server could provide a precise diagnosis of asthma as well as other COPDs [117].

Telemedicine can provide supportive care to asthma patients and help them diagnose the disease without leaving their houses and also predict any probable asthma exacerbations. Systematic and meta-analysis of 2019 found telemedicine could improve asthma control compared to usual care [52]. However, there are multiple challenges regarding the practice of telehealth, for example: (1) it might be difficult for the physician to understand the disease condition better if the patients do not have necessary devices such as thermometer or peak flow meter in their house; (2) the patients might find it troublesome to cope up with a new system; (3) detailed inspection of the throat, nose, and ears to detect upper respiratory symptoms will not be possible; (4) the patients might want to communicate with their providers and refuse to communicate with unfamiliar providers; and (5) many patients might be unaware of this option or not know how to access it [118].

Different online resources from professional societies and regulatory agencies are being developed to overcome such barriers and make people familiar with the telehealth program [116]. Since there is a debate about the increased risk of COVID infection in asthma patients, it is essential to familiarize them with this facility and encourage them to practice it. It is also recommended to keep essential equipment like thermometers and peak flow meters at home. Virtual visits are strongly suggested for patients with uncontrolled asthma and immunodeficiency, but visiting a healthcare facility can be allowed only in extreme situations [28]. As stated by the North American guideline on COVID-19 and contingency planning, "if the allergy/immunology office does not have personal protective equipment (PPE) available, it would be recommended that no patients with a co-potential for an asthma exacerbation and COVID-19 be seen at that office; the patient should instead be seen at another facility capable of COVID-19 isolation which is staffed and equipped to assess and manage asthma" [119].

### Continuation of prescribed medications

Uncontrolled asthma and severe exacerbations can be fatal in the current pandemic. Thus, it is highly recommended to continue taking asthma medications as before, even if the person is suspected to be COVID-19 positive. There is no sufficient data regarding the harmful impacts of consuming asthma drugs or contributions of asthma medications in COVID-19 susceptibility. Besides, several asthma medicines like corticosteroids and bronchodilators are being used in coronavirus treatment [73], suggesting harmless consequences of consuming these medications in asthmatic patients.

### Follow an asthma action plan

Maintaining an asthma action plan can ensure better control of asthma and reduce the need to visit the healthcare facilities amidst this pandemic [120]. CDC suggests everyone with asthma keep an asthma action plan and follow the plan to prevent any severe exacerbations. The action plan might include (i) ensuring all the prescribed drugs and medications are in stock or within the supply, (ii) having a peak flow meter to monitor peak flow rates regularly and predict exacerbations, (iii) avoiding smoking or exposure to second-hand smokes, (iv) reducing anxiety and stress as they can trigger asthma attacks, (v) practicing limited physical activity and breathing exercises, and (vi) continuing medications, including inhalers or steroids, and following the treatment plan accordingly.

### Self-care and management

COVID-19 pandemic has changed our lifestyle and pattern of daily activities. The practice of isolation and lockdown has put a drastic impact on our day-to-day life, as well as our physical and mental health. This sudden change in surroundings can provoke uncontrolled asthma in patients. It has been reported that the most common barriers to asthma self-management include mood disorders and anxiety, social support, and access to health care [52]. Such factors must be taken into consideration to prevent any potential exacerbation during the pandemic.

Potential asthma triggers including allergens, irritants, pet dander, and dust should be avoided as much as possible. Exercising every day can be good practice and may help reduce breathing difficulties, but strenuous exercise should be avoided due to the chance of getting exercise-induced bronchoconstriction. A nutritious and balanced diet can improve overall health and asthma control. A healthy diet plan can also help to maintain body weight, which is essential as asthma tends to be worse in overweight patients. Especially for immune-compromised asthma patients, a well-balanced meal can boost immunity and significantly expand the body's ability to fight against COVID-19 [121].

## Summary and recommendations

The global pandemic caused by SARS-CoV-2 has affected all classes of people from all over the world, but patients with COPD including asthma were presumed to be the worst sufferers. In addition, overlapping representations of asthma and COVID-19 make these conditions difficult to distinguish. However, some unique features of COVID-19 including fever, muscle pain, loss of smell and taste, myalgia, and diarrhea can be used to make differentiate from asthma, but asymptotic COVID-19 patients make it more complex. In addition, wheezing is commonly present in asthma but rarely found in COVID-19 patients. Thus, examining symptoms can play a crucial role in differentiating asthma from COVID-19.

It has been an ongoing debate if asthma patients are at increased risk of getting infected with COVID-19. Asthma patients suppress their immune system by taking different drugs to avoid allergens stimulation. This immune suppression might facilitate viral-induced damage to the airway. The majority of the sources suggest that asthma is not among the risk factors of COVID-19 and might even have a positive influence on this disease susceptibility. However, it should be noted that asthma is under-diagnosed and under-reported due to this pandemic, and also differentiation of symptoms is fierce between the two.

Although there is another debate regarding the possible adverse impact the COVID-19 vaccines may have on asthma patients, no such evidence to such claim is present and it is recommended that in absence of any serious complication or side effects after the first dose everyone must receive the vaccines to ensure protection against the virus.

Different ongoing challenges during the pandemic like shortage of medicines, impact of SDOH, delayed clinical trials are likely to put asthma patients through inconvenience. The primary focus should be to reduce probable asthma attacks as uncontrolled asthma may lead to hospitalization and increase the risk of viral infection. There are also risks regarding the possibility of viral-induced exacerbations, but most of the evidence related to this information comes from human rhinoviruses and no other viruses.

Whether or not to continue consuming asthma medications has been a significant issue as asthma drugs can suppress the airway immune response. There is no certainty to the risks associated with the consumption of such medicines; thus, it is recommended to continue consuming the prescribed medications to manage asthma. However, it is suggested to stop the practice of nebulization, which can increase the risk of viral contamination in both the asthma patients and the bystander hosts. It is also recommended to keep a peak flow meter in the house to diagnose asthma from home and maintain a peak flow diary for better asthma management. Necessary apparatus like thermometers should also be kept in every house so that health care providers can better understand the condition through telehealth facilities. Although there are some barriers to be addressed for better telehealth facilities, most of the organizations are working to reduce such lacking and promote telehealth practice among patients. Moreover, it is recommended that all the clinical trials be shifted to virtual visits, and visiting a hospital should only be considered in extreme conditions.

Management of asthma is the most crucial need during this pandemic. Uncontrolled asthma can be even more dangerous than COVID-19 infection. It is recommended to follow the individualized asthma action plan and to develop self-management interventions to avoid the potential asthma triggers. Additionally, it is extremely important to avoid potential maintain a healthy lifestyle and practice safe hygiene to avoid getting asthma exacerbations from our surroundings and lifestyle choices. A healthy lifestyle and a nutritious diet plan may further boost the immune response to fight off COVID-19 as well as help to manage asthma attacks better.

## Conclusions

COVID-19 has created challenges for regular treatment including asthma. As the COVID-19 is the highest priority and is a threat for other patients to be infected; lockdown, break of the medicine supply chain may cause asthma patients at higher risk. However, there are overlapping respiratory features between asthma and COVID-19, but the modifying effect of asthma in increasing severity of COVID-19 remained unclear. Asthma patients should continue their existing treatment and arrange some small diagnostic devices for a routine check. It is also advised to take telehealth care if necessary, and follow an asthma action plan. Further, self-care and management with a healthy diet and exercise can ensure good immune health of asthma patients during the COVID-19 pandemic.

## Data Availability

Not applicable.

## Abbreviations

COVID-19: Coronavirus disease 2019
SARS-CoV-2: Severe acute respiratory syndrome coronavirus-2
ACE-2: Angiotensin-converting enzyme-2
COPD: Chronic obstructive pulmonary disease
ARDS: Acute respiratory distress syndrome
MERS: Middle East respiratory syndrome
WHO: World Health Organization
CDC: Centers for Disease Control and Prevention
NICE: National Institute for Health and Care Excellence
TLR: Toll-like receptors
S protein: Spike protein
APC: Antigen-presenting cells
IFN: Interferon
IFG: Interferon-stimulated genes
IL: Interleukin
NK: Natural killer cells
DC: Dendritic cells
ICS: Inhaled corticosteroids
FGF: Fibroblast growth factor
G-CSF: Granulocyte-colony stimulating factor
GM-CSF: Granulocyte-macrophage colony-stimulating factor
IP-10: Interferon-γ-inducible protein
MCP-1: Monocyte chemo-attractant protein
MIP-1α: Macrophage inflammatory protein 1 alpha
MIP-1β: Macrophage inflammatory protein 1 beta
PDGF: Platelet-derived growth factor
TNF-α: Tumor necrosis factor
VEGF: Vascular endothelial growth factor
ICU: Intensive care unit
TMPRSS2: Transmembrane protease serine 2 enzyme
AIT: Allergen immunotherapy
SDOH: Social determinants of health
CQ: Chloroquine
HCQ: Hydroxychloroquine
